# Ramadan and gestational diabetes: maternal and neonatal outcomes

**DOI:** 10.1007/s00592-021-01782-y

**Published:** 2021-08-24

**Authors:** Turki Abdullah AlMogbel, Glynis Ross, Ted Wu, Lynda Molyneaux, Maria Ines Constantino, Margaret McGill, Anna Jane Harding, Christine Pech, Abdullah A. Alrasheed, Jencia Wong

**Affiliations:** 1 Sydney Medical School, The University of Sydney, Sydney, Australia; 2 Department of Endocrinology, The RPAH Diabetes Centre, Royal Prince Alfred Hospital, Sydney, Australia; 3 Diabetes and Endocrinology Center, King Fahd Specialist Hospital, Buraydah, Saudi Arabia; 4 Department of Family and Community Medicine, College of Medicine, King Saud University, Riyadh, Saudi Arabia

**Keywords:** Gestational diabetes, Maternal, Neonatal, Outcomes, Pregnancy, Ramadan

## Abstract

**Aims:**

The impact of Ramadan exposure to Gestational Diabetes Mellitus (GDM) pregnancies is not known. We therefore aimed to assess the association of Ramadan with maternal and neonatal outcomes among pregnant women with GDM.

**Methods:**

Retrospective cohort study of 345 Muslim women with singleton pregnancies who attended a major Sydney teaching hospital during the period 1989–2010, was undertaken. Exposure to Ramadan was stratified by the: (1) total pregnancy days exposed to Ramadan, (2) duration (hours) of daily fasting and (3) trimester of exposure. Maternal and neonatal outcomes were examined by exposure status, and never exposed pregnancies were comparator in all three analyses. Fasting status was not recorded.

**Results:**

We found no significant effect of Ramadan exposure on mean birthweight, macrosomia and maternal outcomes. However, we found a significant trend for increased neonatal hyperbilirubinemia with increasing Ramadan days exposure and later trimester exposure (*p*_*trend*_ ≤ 0.02 for both), with adjusted OR 3.9 (*p*=0.03) for those with ≥ 21 days exposure to Ramadan and adjusted OR 4.3 (*p*=0.04) for third trimester exposure. Conversely longer Ramadan exposure and late trimester exposure were independently associated with a lower prevalence of neonatal hypoglycaemia (adjusted OR 0.4 and 0.3 for ≥ 21 days and third trimester exposure, respectively). Furthermore, neonatal hypoglycaemia decreased for the fasting period of > 15 h group (adjusted OR 0.2, *p *= 0.01).

**Conclusions:**

Ramadan exposure is associated with reduced neonatal hypoglycaemia, with no effect on birthweight, implying more favourable glycaemic control. However, the fourfold excess of neonatal hyperbilirubinemia indicates a need for further study of Ramadan and GDM.

**Supplementary Information:**

The online version contains supplementary material available at 10.1007/s00592-021-01782-y.

## Introduction

The Islamic holy month of Ramadan, is one of the five pillars of Islam during which no food or drink is consumed from dawn to sunset for 29–30 days. Gestational diabetes (GDM) has been reported to affect up to 50% of pregnancies in Muslim countries and to date the impact of Ramadan on those with GDM has not been systematically studied [1]. Although International recommendations (with Islamic religious authority approval) state that women with GDM are deemed to be at high risk and are exempted from fasting during Ramadan [2], studies have shown that ~50% of Muslim women with GDM still opt to fast. They do so largely to share the spiritual and social experiences with their family [3, 4]. Even in non-Muslim countries this is so; it has been stated that 54% of pregnant Muslim women living in Western countries reported fasting during Ramadan [5].

It is well recognised that maternal nutrition and lifestyle during pregnancy have important effects both on maternal outcomes and on the health and well-being of the offspring [6]. Ramadan is associated with a number of dietary and lifestyle changes, and the effects of Ramadan may be most significant for those with GDM, which is largely managed by diet and lifestyle [7]. Specifically, Ramadan is associated with an intermittent fasting style of eating pattern, but in addition and irrespective of whether fasting is undertaken, food composition, quantity and physical activity are likely to be changed. Previous studies have shown changes in dietary, physical activity, sleep and diurnal patterns among women during Ramadan [8–11]. Taken together, these Ramadan associated lifestyle changes such as fasting, changes in dietary composition irrespective of fasting, changes in physical activity and circadian rhythm could all potentially and cumulatively affect pregnancy outcomes particularly in the context of GDM.

Studies of the impact of Ramadan have largely studied outcomes in normal pregnancies, and they do vary as to their outcomes. Researchers have studied insulin resistance [12–14], adverse effects on maternal gestational weight gain and infant birthweight [5, 15]. Furthermore, some studies report differing outcomes according to the number of fasting days undertaken [16, 17], and the effects of maternal nutrition on the offspring have been demonstrated to vary according to timing of exposure during the pregnancy [18]. Therefore, in addition to the nutritional, exercise and diurnal patterns seen in Ramadan, there may be differences in effects, dependent on the trimester during which Ramadan falls [5, 19]. We therefore aimed to study the impact of exposure to the holy month of Ramadan, not necessarily fasting behaviour *per se*, on maternal and neonatal outcomes in GDM.

## Methods

This study was a retrospective cohort study of 345 women with GDM. All women with GDM with singleton pregnancies who self-identified as Muslim and attended the Royal Prince Alfred Hospital Antenatal diabetes service during the period 1989–2010 were included in this study. GDM was defined by the Australasian Diabetes in Pregnancy Society (ADIPS) diagnostic criteria, with universal testing between 24 and 28 weeks of gestation implemented since 1991 [20, 21]. Prior to 1989, testing was applied to high risk women only. Maternal and foetal outcomes were available for all study participants. Fasting status was not recorded. Analyses were performed according to the following stratifications and methods:

Firstly, the main study sample (*n* = 345) was grouped according to the number of *days* of the pregnancy which coincided with the month of Ramadan (number of days as a measure of “exposure”–Ramadan); No exposure (*n* = 48); 1–10 days exposure (*n* = 17); 11–20 days exposure (*n* = *23*) and 21–30 days exposure (*n* = 257).

To examine the impact of the *duration of Ramadan fasting per day*, firstly in order to standardise the days of exposure, the cohort were restricted to include only pregnancies who had been exposed to the entire month of Ramadan (*n* = 245).The variation in fasting duration occurs due to the fact that Ramadan is determined by the lunar (Islamic; Arabic) calendar and occurs 10–11 days earlier every year in comparison to the Gregorian (western) calendar [2]. Thus, over time Ramadan falls on different seasons with different time periods between dawn and sunset which determines the daily fasting duration. The fasting duration for any given year can be ascertained from the Islamic calendar specific to Sydney. In our study, data collection took place over a 22 year period, and accordingly, fasting duration varied from ~12 h (in 2010 and 1989) to 16 h (in 1998–2001). Those not exposed to Ramadan during pregnancy were used as comparator (*n* = 48).

To examine the impact of the *trimester* during which exposure Ramadan occurred, we excluded women for whom Ramadan exposure occurred during overlapping trimesters (*n* = 107) resulting in an analysis set of 238 pregnancies. The groups were stratified by no trimester exposure to Ramadan (*n* = 48); first trimester exposure only (*n* = 59); second trimester exposure only (*n* = 75); third trimester exposure only (*n* = 56).

Demographic variables collected included ethnicity, family history of diabetes, parity, pre-gestational weight and gestation at diagnosis of GDM. Maternal outcomes included gestational weight gain, insulin use, hypertensive disorders of pregnancy (defined as pre-eclampsia or systolic blood pressure ≥ 140 mmHg and/or diastolic blood pressure ≥ 90 mmHg in a previously normotensive pregnant women who were at 20 weeks or more of pregnancy and had no proteinuria or new signs of end-organ dysfunction), gestation weeks at confinement, pre-term delivery (defined as delivery before 37 weeks of gestation) and caesarean section. Neonatal outcomes were assessed by birthweight, large for gestational age (LGA), small for gestational age (SGA) (both defined as according to gender- and gestational age-specific birthweight > 90th centile and < 10th centile, respectively, for New South Wales, Australia, population) [22] and macrosomia (defined as a birthweight of 4000 g or more); neonatal hyperbilirubinemia (defined by clinician-determined need for phototherapy or exchange transfusion) [23]; and neonatal hypoglycaemia (defined as blood glucose level (BGL) < 2.5 mmol/L, detected at any stage post-partum and routinely checked within 24 h post-partum in all offspring of women with GDM).

Data were analysed using NCSS 2007. Continuous data were checked for normality and presented as mean or median. Data not normally distributed were transformed for analysis. ANOVA or Kruskal–Wallis tests were used to compare means or medians. The post hoc tests Bonferroni or Kruskal–Wallis *Z* tests were used to adjust for multiple comparisons when significant. *T* *trend* test was also generated for suspected trends. Categorical data were presented as a percentage. Chi-square was used to compare groups. No exposure was nominated as the reference group for all analysis. All maternal and neonatal outcomes were also assessed according to the above stratifications. Logistic regression was used to determine the neonatal outcomes according to the degree of exposure to Ramadan and adjusted for significant confounders. Odds ratios (OR) were generated for each neonatal outcome according to the stratifications. Armitage proportion trend test was calculated to determine any trend for the neonatal outcomes according to Ramadan exposure. Multiple regression was used to determine baby weight difference grouped by Ramadan exposure. Statistical significance was accepted at *p* < 0.05. The study protocol was approved by Sydney Local Health District Ethics Review Committee (RPAH Zone).

## Results

The demographic characteristics of the women not exposed to Ramadan (*n* = 48) and those having had any exposure to Ramadan (*n* = 297) are shown in Supplementary Table 1. No significant differences were found in ethnicity, age at pregnancy, pre-gestational BMI, family history of diabetes or parity between the groups.

### Days of exposure to Ramadan

Table 1 shows the results of analysis grouped by days of exposure to Ramadan (*n* = 345). There were no differences in maternal and neonatal outcomes. However, differences were observed in neonatal complications. There was a trend for an increasing prevalence of neonatal hyperbilirubinemia with increasing days of exposure to Ramadan (p_*trend*_ = 0.004). The prevalence of neonatal hyperbilirubinemia was 6.8% in the group never exposed to Ramadan and 22.8% for the highest days exposure group with a significant difference between these two groups (*p* = 0.01). When this outcome was examined by logistic regression, the OR for hyperbilirubinemia was 3.9 (95% CI 1.1–13.1, *p* = 0.03) for the group with the greatest exposure. (Table 2). Conversely, there was a trend towards a lower prevalence of neonatal hypoglycaemia with increasing days of exposure to Ramadan: a prevalence of 22.7% for the no exposure group and 10.4% in the highest exposure group (p_*trend*_ = 0.05 across the groups). The prevalence of neonatal hypoglycaemia among those exposed to > 10 days of Ramadan was significantly different to the no exposure group (Table 1). Furthermore, when examined by logistic regression, OR for neonatal hypoglycaemia was significantly reduced to 0.4 (95% CI 0.2–0.9, *p* = 0.02) for the longest exposure group, with the no exposure group as reference (Table 2).

**Table 1:** The outcomes stratified by days of exposure to Ramadan

*N* = 345		No exposure to the whole of Ramadan (*n* = 48)	1–10 days of pregnancy exposed to Ramadan (*n* = 17)	11–20 days of pregnancy exposed to Ramadan (*n* = 23)	21–30 days of pregnancy exposed to Ramadan (*n* = 257)	*P* value
*Maternal demographics*						
Ethnicity (%):	Arab	35.4	23.5	34.8	43.2	0.6
	Indian	52.1	52.9	39.1	40.5	
	Asian	10.4	11.8	13	8.9	
	Other	2.1	11.8	13	7.4	
Maternal age (years)		30.8 ± 4.5	31.4 ± 4.2	32.2 ± 5.2	31.2 ± 5.3	0.8
Pre-pregnancy BMI (kg/m^2^)		25.2 ± 4.3	27.1 ± 6.9	24.3 ± 4.3	26.5 ± 5.7	0.2
Family history of DM (%)		67.4	58.8	47.8	61.7	0.5
Gestational age at diagnosis of GDM (weeks)		24.6 ± 7.9	25.1 ± 7.1	25.5 ± 6.6	25.5 ± 6.4	0.9 T_*trend*_ p = 0.4
*GDM diagnosis and intervention*						
OGTT results:	0 min	4.8 ± 0.6	5.0 ± 0.7	5.1 ± 0.9	5.0 ± 0.9	0.4
	60 min	9.9 ± 1.5	10.1 ± 1.7	11.0 ± 2.0	10.3 ± 1.7	0.1
	120 min	8.4 ± 1.6	9.2 ± 2.0	9.1 ± 2.0	8.6 ± 1.7	0.3
	AUC	992 ± 118	1032 ± 167	1062 ± 206	1017 ± 158	0.4
Insulin treatment (%)		60.4	50	60.9	56.8	0.7
Gestation insulin commenced (weeks)		28.0 ± 6.6	23.1 ± 10.9	28.5 ± 6.2	28.6 ± 6.7	0.2
Maximum insulin dose (units)		34 [18–54]	40 [18–66]	24 [6–61]	34 [16–63]	0.6
*Pregnancy outcomes*						
Gestation at confinement (weeks)		38.2 ± 2.9	38.6 ± 1.1	39.2 ± 1.3	38.5 ± 1.8	0.2
Weight gain during pregnancy (kg)		12.2 ± 6.3	12.7 ± 5.6	12.0 ± 5.0	13.0 ± 5.2	0.8
Pre-term delivery (< 37 weeks) (%)		8.3	6.3	0	10.7	Z_*trend*_ *p* = 0.2
Caesarean section (%)		29.8	46.7	34.8	35.4	0.7
Gestational HTN (%)		14.6	23.5	0	15.2	*p* = 0.6
Birthweight (grams)		3140 ± 619	3244 ± 293	3263 ± 696	3250 ± 569	*p* = 0.7
Macrosomia (%)		4.3	0	8.7	9.1	Z_*trend*_ *p* = 0.2
LGA (%)		6.4	0	4.3	9.6	Z_*trend*_ *p* = 0.2
SGA (%)		12.8	0	13.0	5.6	Z_*trend*_ *p* = 0.1
Stillbirth (%)		2.1	0	0	0.4	N/A
Neonatal death (%)		0	0	0	0.3	N/A
Neonatal hypoglycaemia (%)		22.7	26.7	0**	10.4^¶^	Z_*trend*_ *p* = 0.05
Neonatal hyperbilirubinemia (%)		6.8	7.1	13.0	22.8**	Z_*trend*_ *p* = **0.004**
*Maternal post-partum glycaemic status*						
Post-delivery GTT (%)	No diabetes	90.9	80	75	78.6	N/A
	IGT	4.5	20	16.7	17.9	
	Diabetes	4.5	0	8.3	3.6	

Data are %; mean ± SD; or median [IQR]

**p ≤ 0.01 for comparison with No exposure group as comparator

^¶^*p* < 0.05 for comparison with No exposure group as comparator

**Table 2:** Relationship between Ramadan exposure in GDM and neonatal outcomes

Ramadan exposure measure		Neonatal hypoglycaemia			Neonatal hyperbilirubinemia		
		OR	95% CI	*P*	OR	95% CI	*P*
Days exposure analysis	Model 1*				Model 1*		
	Never exposed	Ref			Ref		
	1–10 days	1.2	0.3–4.7	0.8	1.1	0.1–11.0	0.9
	11–20 days	0.1	0–100	0.9	2.1	0.4–11.1	0.4
	21–30 days	0.4	0.2–0.9	**0.03**	4.0	1.2–13.5	**0.02**
	Model 2#				Model 2§		
	Never exposed	Ref			Ref		
	1–10 days	1.4	0.3–5.2	0.7	1.0	0.09–10.9	1.0
	11–20 days	0.1	0.–100	0.9	2.4	0.4–13.3	0.3
	21–30 days	0.4	0.2–0.9	**0.02**	3.9	1.1–13.1	**0.03**
	Model 3**						
	Never exposed	Ref					
	1–10 days	1.4	0.3–5.2	0.6			
	11–20 days	0.1	0.–100	0.9			
	21–30 days	0.4	0.2–0.8	**0.02**			
Hours exposure analysis (fasting period /day)	Model 1*				Model 1*		
	Never exposed	Ref			Ref		
	> 12–13 h	0.4	0.09–1.5	0.2	4.8	1.1–19.7	**0.03**
	> 13–14 h	0.4	0.2–1.2	0.1	7.0	2.0–24.6	**0.003**
	> 14–15 h	0.7	0.2–1.9	0.4	2.7	0.6–11.0	0.2
	> 15–16 h	0.3	0.09–0.8	**0.02**	2.2	0.6–8.5	0.2
	Model 2#				Model 2§		
	Never exposed	Ref			Ref		
	> 12–13 h	0.3	0.08–1.3	0.1	3.9	0.9–16.5	0.06
	> 13–14 h	0.4	0.2–1.1	0.09	6.8	1.9–24.3	**0.003**
	> 14–15 h	0.7	0.2–1.9	0.4	2.7	0.6–11.4	0.2
	> 15–16 h	0.2	0.08–0.7	**0.01**	2.2	0.6–8.3	0.3
	Model 3**						
	Never exposed	Ref					
	> 12–13 h	0.3	0.08–1.3	0.1			
	> 13–14 h	0.4	0.2–1.1	0.09			
	> 14–15 h	0.6	0.2–1.9	0.4			
	> 15–16 h	0.2	0.08–0.7	**0.01**			
Trimester exposure analysis	Model 1*				Model 1*		
	Never exposed	Ref			Ref		
	1st Trimester	0.4	0.1–1.3	0.1	2.7	0.7–10.6	0.2
	2nd trimester	0.3	0.1–0.9	**0.04**	4.1	1.1–15.1	**0.03**
	3rd trimester	0.3	0.1–1.1	0.06	4.6	1.2–17.0	**0.02**
	Model 2#				Model 2¶		
	Never exposed	Ref			Ref		
	1st trimester	0.4	0.1–1.1	0.07	2.2	0.5–9.2	0.3
	2nd trimester	0.3	0.1–0.9	**0.03**	4.2	1.1–15.8	**0.03**
	3rd trimester	0.3	0.09–1.03	0.06	4.3	1.1–16.6	**0.04**
	Model 3**						
	Never exposed	Ref					
	1st trimester	0.4	0.1–1.1	0.07			
	2nd trimester	0.3	0.1–0.9	**0.03**			
	3rd trimester	0.3	0.09–1.01	0.05			

*Unadjusted model

§Adjusted for gestational age at delivery, gender, ethnicity and birthweight centile

¶Adjusted for gestational age at delivery, gender, ethnicity, birthweight centile and gestational age at diagnosis

#Adjusted for gestational age at delivery, insulin treatment and birthweight centile

**Adjusted for gestational age at delivery, insulin treatment and LGA

### Duration of the Ramadan fasting period

Table 3 presents the outcomes stratified by the duration of the fasting period measured in hours during Ramadan. We observed a trend for the mean birthweight to increase as the fasting period/day increased (*p*_*trend*_ = 0.02). A similar pattern was seen for the weight outcomes of macrosomia and LGA although this was not statistically significant (*p*_*trend*_for both = 0.1). Conversely, the highest prevalence of SGA was seen in the non-Ramadan exposed (*p*_*trend*_ = 0.03). However, there was no significant relationship between hours of fasting and birthweight in multivariate model (Supplementary Table 3).

**Table 3:** The outcomes stratified by the duration of the fasting period measured in hours during Ramadan

*N* = 293		No exposure to the whole of Ramadan (*n* = 48)	Fasting period/day > 12–13 h (*n* = 31)	Fasting period/day > 13–14 h (*n* = 85)	Fasting period/day > 14–15 h (*n* = 43)	Fasting period/day > 15–16 h (*n* = 86)	*P* value
*Maternal demographics*							
Ethnicity (%):	1. Arab	35.4	22.6	40	48.8	51.2	0.08
	2.Indian	52.1	54.8	44.7	44.2	29.1	
	3.Asian	10.4	9.7	7.1	4.7	12.8	
	4.Other	2.1	12.9	8.2	2.3	7	
Maternal age (years)		30.8 ± 4.5	31.6 ± 5.6	31.6 ± 5.1	29.9 ± 5.0	31.4 ± 5.6	0.3
Pre-pregnancy BMI (kg/m^2^)		25.2 ± 4.3	27.0 ± 4.8	26.1 ± 6.0	26.7 ± 5.7	26.6 ± 5.8	0.6
Family history of DM (%)		67.4	61.3	56.6	55.8	69	0.4
Gestation age at diagnosis of GDM (weeks)		24.6 ± 7.9	23.8 ± 7.5	24.4 ± 6.0	25.7 ± 6.1	26.5 ± 6.3	0.2
*GDM diagnosis and intervention*							
OGTT results:	0 min	4.8 ± 0.6	5.4 ± 1.3	5.0 ± 0.9	5.2 ± 0.9	4.8 ± 0.8	**0.002**
	60 min	9.9 ± 1.5	10.8 ± 2.0	10.3 ± 1.8	10.3 ± 1.5	10.0 ± 1.6	0.1
	120 min	8.4 ± 1.6	8.8 ± 2.3	8.6 ± 1.8	8.7 ± 1.5	8.6 ± 1.4	0.9
	AUC	992 ± 118	1058 ± 223	1018 ± 121	1034 ± 121	988 ± 134	0.2
Insulin treatment (%)		60.4	61.3	68.2	58.1	40.7	**0.007** Z_*trend*_ *p* = **0.004**
Gestation insulin commenced (weeks)		28.0 ± 6.6	26.5 ± 5.8	27.8 ± 6.8	28.6 ± 6.1	31.0 ± 6.4	0.3
Maximum insulin dose (units)		34[18–54]	38[16–54]	39[19–73]	27[21–50]	32[13–80]	0.8
*Pregnancy outcomes (short-term)*							
Gestation at confinement (weeks)		38.2 ± 2.9	38.0 ± 1.6	38.5 ± 2.1	38.8 ± 1.4	38.7 ± 1.7	0.3
Weight gain during pregnancy (kg)		12.2 ± 6.2	12.0 ± 6.3	13.0 ± 3.7	12.3 ± 4.3	12.4 ± 5.7	0.9
Pre-term delivery (< 37 weeks) (%)		8.3	9.7	12	9.3	9.6	N/A
Caesarean section (%)		29.8	48.4	45.8	30.2	27.1	**0.04** Z_*trend*_ *p* = 0.1
Gestational HTN (%)		14.6	25.8	14.1	14	15.1	0.3
Birthweight (grams)		3140 ± 619	3107 ± 521	3197 ± 598	3265 ± 488	3347 ± 584	0.2 T_*trend*_ *p* = **0.02**
Macrosomia (%)		4.3	6.5	7.2	7	11.8	Z_*trend*_ *p* = 0.1
LGA (%)		6.4	6.5	7.2	9.3	13.3	Z_*trend*_ *p* = 0.1
SGA (%)		12.8	3.2	12	0**	3.6	Z_*trend*_ *p* = **0.03**
Deceased birth/stillbirth (%)		2.1	0	1.2	0	0	N/A
Deceased post-partum/neonatal death (%)		0	0	1.2	0	0	N/A
Neonatal hypoglycaemia (%)		22.7	9.7	11.3	16.3	7.2**	Z_*trend*_ *p* = 0.06
Neonatal hyperbilirubinemia (%)		6.8	25.8**	33.8**	16.3	14.3	Z_*trend*_ *p* = 0.6
*Maternal post-partum glycaemic status*							
Post-delivery GTT (%)	No diabetes	90.9	87.5	73.3	77.8	77.3	N/A
	IGT	4.5	6.3	20	22.2	20.5	
	Diabetes	4.5	6.3	6.7	0	2.3	

*Data are %; mean ± SD; or median [IQR]

**p < 0.05 for comparison with no exposure group as comparator

The need for insulin was significantly different across the duration groups (*p* = 0.007); insulin use ranged from 40 to 68%, but the lowest proportion of insulin use was seen in the highest duration group (*p*_*trend*_ = 0.004). We observed that the prevalence of neonatal hypoglycaemia was lowest in those in the longest duration group compared to those never exposed (*p* = 0.02). Furthermore, the adjusted OR for neonatal hypoglycaemia was decreased for the group where the fasting period was > 15 h (OR 0.2, 95% CI 0.08–0.7; *p* = 0.01) (Table 2).

### Trimester of exposure to Ramadan

There were no differences in maternal age, ethnicity and pre-pregnancy BMI between the groups (Table 4). As would be expected, differences in gestational age at GDM diagnosis and family history of diabetes were seen (*p* < 0.05 for both). There was a significant trend towards increasing birthweight by later trimester exposure (*p*_trend_ = 0.03). Furthermore, there were no relationships between LGA, SGA or mean birthweight and trimester exposure (Supplementary Tables 2and 3).

**Table 4:** The outcomes stratified according to Ramadan time passed in pregnancy (*N* = 238)

		No exposure to Ramadan (*n* = 48)	1st trimester only exposure to Ramadan (*n* = 59)	2nd trimester only exposure to Ramadan (*n* = 75)	3rd trimester only exposure to Ramadan (*n* = 56)	*P* value
*Maternal demographics*						
Ethnicity (%):	1. Arab	39.2	37.5	45.6	48.1	0.6
	2.Indian	49	44.6	35.4	38.9	
	3.Asian	9.8	7.1	10.1	9.3	
	4.Other	2	10.7	8.9	3.7	
Maternal age (years)		30.8 ± 4.5	31.4 ± 5.3	32.2 ± 4.8	30.2 ± 5.9	0.2
Weight gain during pregnancy (kg)		12.2 ± 6.3	8.9 ± 7.7	12.0 ± 5.8	12.5 ± 5.2	0.1
Pre-pregnancy BMI (kg/m^2^)		25.2 ± 4.3	26.6 ± 6.2	27.5 ± 5.0	26.0 ± 5.3	0.1
Family history of DM (%)		67.3	75.9	55.1	51.9	**0.03** Z_*trend*_ *p* = **0.02**
Gestation age at diagnosis of GDM (weeks)		24.6 ± 7.9	23.9 ± 6.4	23.9 ± 6.7	27.2 ± 6.2	**0.04** T_*trend*_ *p* = 0.07
*GDM diagnosis and intervention*						
OGTT results:	0 min	4.8 ± 0.6	5.2 ± 1.3	4.9 ± 0.8	5.0 ± 0.8	0.2
	60 min	9.9 ± 1.5	10.7 ± 2.1	9.9 ± 1.6	10.5 ± 1.7	**0.046**
	120 min	8.4 ± 1.6	9.0 ± 2.1	8.6 ± 1.4	8.6 ± 1.7	0.3
	AUC	993 ± 118	1067 ± 205	994 ± 130	1018 ± 166	0.05
Insulin treatment (%)		60.4	55.9	60	53.6	0.9 Z_*trend*_ *p* = 0.6
Gestation insulin commenced (weeks)		28.0 ± 6.7	27.6 ± 5.4	28.2 ± 7.7	29.0 ± 5.7	0.9
Maximum insulin dose (units)		34 [18–54]	32 [22–60]	34 [20–63]	47 [14–78]	0.8
*Pregnancy outcomes (short-term)*						
Gestation at confinement (weeks)		38.1 ± 2.9	38.5 ± 1.5	38.7 ± 1.5	38.8 ± 1.4	0.06
Pre-term delivery (< 37 weeks) (%)		8.3	10.5	8.2	7.1	0.9 Z_*trend*_ *p* = 0.7
Caesarean section (%)		29.8	38.6	33.8	33.9	0.8 Z_*trend*_ *p* = 0.9
Gestational HTN (%)		14.6	15.3	17.3	14.3	1.0 Z_*trend*_ *p* = 1.0
Baby weight (grams)		3140 ± 619	3163 ± 603	3272 ± 523	3351 ± 497	0.2 T_*trend*_ *p* = **0.03**
Macrosomia (%)		4.3	7.0	9.5	5.4	Z_*trend*_ *p* = 0.7
LGA (%)		6.4	7.0	6.9	10.7	*p* = 0.8 Z_*trend*_ *p* = 0.4
SGA (%)		12.8	7.0	4.2	7.1	*p* = 0.4 Z_*trend*_ *p* = 0.3
Deceased birth/stillbirth (%)		2.1	0	0	1.8	N/A
Deceased post-partum/neonatal death (%)		0	0	0	1.9	N/A
Neonatal hypoglycaemia (%)		22.7	10.9	8.3	8.9	*p* = 0.09 Z_*trend*_ *p* = **0.048**
Neonatal hyperbilirubinemia (%)		6.8	16.4	23.3	25.0	*p* = 0.08 Z_*trend*_ *p* = **0.02**
*Maternal post-partum glycaemic status*						
Post-delivery GTT (%)	No diabetes	90.9	69.2	76.9	72	N/A
	IGT	4.5	23.1	23.1	28	
	Diabetes	4.5	7.7	0	0	

*Data are %; mean ± SD; or median [IQR]

There was a significant trend towards increasing neonatal hyperbilirubinemia by later trimester exposure (Table 4, p_*trend*_ = 0.02), and the OR for hyperbilirubinemia was 4.2 (1.1–15.8, *p* = 0.03) and 4.3 (1.1–16.6, *p*=0.04, Table 2) for second and third trimester exposure, respectively, after adjustment for gestational age at delivery, gestation at diagnosis, ethnicity, gender and percentile birthweight. Later trimester exposure to Ramadan was associated with a lower prevalence of neonatal hypoglycaemia (p_*trend*_ = 0.05). The adjusted OR for neonatal hypoglycaemia was 0.4, 0.3 and 0.3 for 1st, 2nd and 3rd trimester exposure, respectively (*p* = 0.07; *p* = 0.03; *p* = 0.05, respectively), after adjustment for insulin use, gestational age and LGA (Table 2).

## Discussion

In this retrospective, cohort study spanning over many years and seasons; we examined three aspects of Ramadan exposure on pregnancy outcomes in the context of GDM. Importantly, we found no significant independent association between the number of days of exposure to Ramadan or the trimester of exposure on mean birthweight, macrosomia, LGA or SGA. An increased fasting period was associated with increasing birthweight (p_*trend*_ = 0.02); however, adjustment for known risk factors attenuated this relationship. Importantly, we did observe an increased prevalence of neonatal hyperbilirubinemia with an increasing number of days exposure to Ramadan and by later trimester exposure also with an ~fourfold increase in the adjusted odds of hyperbilirubinemia observed for exposure duration of > 21 days of the pregnancy and also exposure in the last trimester. The potential duration of daily fasting had no impact on this outcome. Conversely, greater exposure to Ramadan in terms of days and later trimester exposure were separately associated with a lower prevalence of neonatal hypoglycaemia even after correcting for known confounders. A fasting period of > 15 h/day was also associated with less neonatal hypoglycaemia. Reassuringly, no differences were seen in maternal weight gain, insulin use, pre-term delivery or stillbirth and any of the exposure parameters.

As previously mentioned, there is evidence that Ramadan may have effects on birthweight although the literature is not consistent. An Iranian study showed that neonatal birthweight in those fasting in pregnancy was 100 g more than those of the non-fasting group (*p* = 0.009) [24]. Similarly, a study of 4343 women with singleton pregnancies showed that mean birthweight was higher in the group fasting for more than 20 days in comparison with the non-fasting group or to those with a shorter number of days fasting (*p* = 0.0006) [16]. Alternatively, Awwad et al [15] found that mean birthweight was lower in the Ramadan-fasted women (*p*=0.024), and this study excluded those with maternal diabetes; similarly, a small multiethnic study from the Netherlands showed that newborns of mothers who fasted more than half a month were lighter than those of mothers who did not fast, but this was not a statistically significant finding [5]. Although we determined an initial positive relationship between birthweight and duration of fasting and late trimester exposure, the systematic data collection allowed the appropriate adjustments for most confounding variables which explained the observations. It is possible also that the relationships observed between birthweight and fasting duration can be explained by previously reported seasonal and high temperature effects on birthweight [25]. Furthermore, it is important to note that in the context of our study cohort with GDM, dietary advice and indeed insulin treatment were also offered to all women and this may have attenuated any effects of Ramadan exposure on birthweight. Regardless of this, it is reassuring to at least observe no significant adverse effects on LGA or SGA in treated GDM.

Of concern is our finding that the rate of neonatal hyperbilirubinemia was 22.8% in the group with the greatest days of exposure to Ramadan as compared to 6.8% of the never exposed group, and there was a significant dose-dependent association between days of exposure and hyperbilirubinemia. This is particularly important, given that the diagnosis is largely made on clinical grounds in our unit and thus clinically significant. Physiological jaundice is due to the developmental insufficiency in the disposition of bilirubin within newborn liver. Reduced gut motility, an absence of intestinal flora that degrade bilirubin and a delay in the excretion of bilirubin rich meconium, may all contribute to hyperbilirubinemia by increasing enterohepatic circulation. However, the excess of hyperbilirubinemia seen here in association with Ramadan is largely unexplained and has not previously been examined or reported in pregnancy studies of Ramadan or fasting. We have tried to account for potential contributors in regression analysis, but there may be some residual confounding not accounted for, e.g. G6PD deficiency. Our findings are of clinical concern given that neonatal jaundice is the most common cause of hospitalisation in the immediate post-natal period.

On the other hand, all the strata of Ramadan exposure; the higher number of days exposed, late trimester exposure and a long fasting duration appeared to be somewhat protective of neonatal hypoglycaemia. This is without significant changes in LGA and macrosomia risk, and the observations remained same after adjustments. The inference here is that those with the greater exposure to Ramadan, especially closer to delivery in the third trimester and a long duration of fasting may have better glycaemic control, less foetal hyperinsulinemia and therefore, less hypoglycaemia risk. These observations are hypothesis generating only but the evidence suggests a need for further study in GDM. In a recent study using CGM, Ramadan fasting in women with GDM treated with diet alone or with diet plus metformin was associated with lower mean glucose levels which would be consistent with our findings [26]. This study, however, detected higher rates of hypoglycaemia in women with GDM, and authors advised against fasting during Ramadan until further data are available.

There is a paucity of data on the association of Ramadan and pregnancy outcomes in a large GDM cohort, and this is the first study that has been able to examine the impact according to three different aspects of Ramadan exposure. One of the strengths of this study is the ability to examine the associations between the seasonal variations in fasting duration, made possible by the long duration over which the data have been collected. Another strength of this study is the standardised and systematised prospective data collection and standardised management practise in a single centre over 22 years. This allows for more robust comparison of exposure groups with minimal effects of treatment confounding [27]. Further, the novelty of the study is provided by examining the neonatal outcomes of neonatal hypoglycaemia and neonatal hyperbilirubinemia as a Ramadan exposure outcome, not previously reported on. The ability to have extensive contemporaneously collected clinical data to account for many confounders has also been of benefit.

A limitation of this study is not knowing specifically which patient had fasted and for how long over their pregnancy. Thus, whether our findings are specific to fasting per se or the overall altered dietary, exercise or diurnal patterns seen in Ramadan irrespective of fasting or residual unmeasured confounders cannot be determined. Another limitation of our study is that information was not available on how hypoglycaemic therapy had been managed during Ramadan, and this could influence glycaemic control and consequently neonatal outcomes. Furthermore, although our treatment protocols for glycaemic management are standardised, we cannot account for any changes in glycaemic control between the exposure groups which may have modified our outcomes.

In conclusion, we could not demonstrate any independent adverse effect of Ramadan exposure, either by the quantity of days exposed, trimester of exposure or length of fasting period on birthweight or gestational weight gain. Our data suggest a protective effect of Ramadan exposure on neonatal hypoglycaemia, potentially by improved glycaemia. Nevertheless, our finding of an excess in neonatal hyperbilirubinemia remains unexplained and of concern. With the increasing rates of GDM in Muslim countries and an understanding that many women still choose to fast despite dispensations, we believe our data support the need for a robust prospective study of the neonatal effects of Ramadan on GDM pregnancies. These findings support the current guidelines for women with GDM to avoid fasting during Ramadan until further evidence is available.

## Supplementary Information

Below is the link to the electronic supplementary material.

Supplementary file1 (PDF 209 KB)

## Data Availability

Data are available from corresponding author on request.
