# Supplementary material for: Ramadan and gestational diabetes: maternal and neonatal outcomes
Source: Acta Diabetol. 2021 Aug 24;59(1):21–30. doi: 10.1007/s00592-021-01782-y (PMC8758603; doi:10.1007/s00592-021-01782-y)
Supplement: Supplementary file 1 — Supplementary file1 (PDF 209 KB) [file 592_2021_1782_MOESM1_ESM.pdf]

Supplementary Table1: The demographic characteristics of the women not exposed to Ramadan (n=48) and those having had any exposure to Ramadan (n=297).*

N= 345		All Ramadan coincided outside the pregnancy (n=48)	Ever exposed to Ramadan (any days coincided ≥1) (n=297)	P value
**Maternal Demographics**				
Ethnicity (%):	1.Arab	35.4	41.4	p=0.5
	2.Indian	52.1	41.1	
	3.Asian	10.4	9.4	
	4.Other	2.1	8.1	
Age at pregnancy (years)		30.8 ±4.5	31.3 ± 5.2	p=0.5
Initial Weight (kg.)		64.2 ± 10.9	66.3 ± 14.6	p=0.4
Final Weight (kg.)		75.6 ± 10.5	78.8 ± 14.2	p=0.1
Weight gain during pregnancy (kg)		12.2 ± 6.3	12.2 ± 5.5	p=1.0
Pre pregnancy BMI (kg/m^2)		25.2 ± 4.3	26.4 ± 5.7	p=0.2
Family History (%)		67.3	59.0	p=0.3
Parity(%):				p=0.5
0 (Primip)		44.0	36.5	
1		22.0	28.0	
≥2 (Multip)		34.0	35.5	
**GDM Diagnosis & Intervention**				
OGTT Results:	0 minutes	4.8 ± 0.6	5.0 ± 0.9	p=0.2
	60 minutes	10.0 ± 1.5	10.3 ± 1.7	p=0.2
	120 minutes	8.4 ± 1.5	8.7 ± 1.6	p=0.3
	AUC	995 ± 117	1017 ± 154	p=0.4
Gestation at diagnosis of GDM (weeks)		24.3 ± 7.8	25.5± 6.3	p=0.2
Insulin Treatment (%)		56.9	56.5	p=0.9
Gestation Insulin commenced (weeks)		28.9± 5.0	28.4 ± 6.4	p=0.7
Maximum insulin dose (units)		34[18-54]	34[16-62]	P=0.9

*Data are %; mean ± SD; or median [IQR].

Supplementary Table 2: Relationship between Ramadan exposure in GDM and Neonatal outcomes LGA and SGA

Analysis		LGA			SGA		
		OR	95%CI	*P*	OR	95%CI	*P*
**Days exposure analysis**	Model 1*				Model 1*		
	Never exposed to Ramadan	Ref.			Ref.		
	1–10 days exposure	0.1	0-100	0.9	0.1	0-100	0.9
	11-20 days exposure	0.7	0.07-6.8	0.7	1.0	0.2-4.5	0.9
	21-30 days exposure	1.6	0.4-5.3	0.5	0.4	0.1-1.1	0.08
	Model 2¶				Model 2¶		
	Never exposed to Ramadan	Ref.			Ref.		
	1–10 days exposure	0.1	0-10	1.0	0.1	0-10	0.7
	11-20 days exposure	0.6	0.06-6.4	0.7	1.0	0.2-4.4	1.0
	21-30 days exposure	1.6	0.5-5.6	0.5	0.4	0.1-1.1	0.07
**Hours exposure (duration of daily fasting) analysis**	Model 1*				Model 1*		
	Never exposed to Ramadan	Ref.			Ref.		
	Fasting period/day >12 – 13 hours	1.0	0.2-6.4	1.0	N/A	N/A	N/A
	Fasting period/day >13 – 14 hours	1.1	0.3-4.8	0.9	N/A	N/A	N/A
	Fasting period/day >14 – 15 hours	1.5	0.3-7.1	0.6	N/A	N/A	N/A
	Fasting period/day >15 – 16 hours	2.2	0.6-8.5	0.2	N/A	N/A	N/A
	Model 2§				Model 2§		
	Never exposed to Ramadan	Ref.			Ref.		
	Fasting period/day >12 – 13 hours	0.4	0.02-5.1	0.4	N/A	N/A	N/A
	Fasting period/day >13 – 14 hours	0.6	0.08-3.9	0.6	N/A	N/A	N/A
	Fasting period/day >14 – 15 hours	2.8	0.4-18.2	0.3	N/A	N/A	N/A
	Fasting period/day >15 – 16 hours	1.1	0.2-6.2	0.9	N/A	N/A	N/A
**Trimester exposure analysis**	Model 1*				Model 1*		
	Never exposed to Ramadan	Ref.			Ref.		
	1st Trimester exposure	1.1	0.2-5.2	0.9	0.5	0.1-1.9	0.3
	2nd Trimester exposure	1.1	0.2-4.8	0.9	0.3	0.07-1.3	0.1
	3rd Trimester exposure	1.8	0.4-7.5	0.4	0.5	0.1-2.0	0.3
	Model 2§				Model 2§		
	Never exposed to Ramadan	Ref.			Ref.		
	1st Trimester exposure	0.5	0.05-4.7	0.5	0.6	0.1-2.6	0.5
	2nd Trimester exposure	0.7	0.09-5.1	0.7	0.2	0.04-1.2	0.08
	3rd Trimester exposure	1.8	0.3-12.0	0.5	0.5	0.1-2.3	0.4

* Unadjusted model

¶ Adjusted for gestational age at delivery, gender, ethnicity, maternal age, and hypertensive disorders of pregnancy.

§Adjusted for gestational age at delivery, gender, ethnicity, maternal age, mother weight gain, patient weight before pregnancy, and hypertensive disorders of pregnancy.

Supplementary Table 3: Relationship between Ramadan exposure in GDM and Birth weight

Analysis		Birth weight	
		T value	*P*
**Days exposure analysis**	Model 1*		
	Never exposed to Ramadan	Ref.	
	1–10 days exposure	0.6	0.5
	11-20 days exposure	0.8	0.4
	21-30 days exposure	1.2	0.2
	Model 2§		
	Never exposed to Ramadan	Ref.	
	1–10 days exposure	0.9	0.3
	11-20 days exposure	-0.3	0.8
	21-30 days exposure	0.5	0.6
**Hours exposure (duration of daily fasting) analysis**	Model 1*		
	Never exposed to Ramadan	Ref.	
	Fasting period/day >12 – 13 hours	-0.2	0.8
	Fasting period/day >13 – 14 hours	0.5	0.6
	Fasting period/day >14 – 15 hours	1.0	0.3
	Fasting period/day >15 – 16 hours	2.0	**0.05**
	Model 2§		
	Never exposed to Ramadan	Ref.	
	Fasting period/day >12 – 13 hours	0.1	0.9
	Fasting period/day >13 – 14 hours	-0.3	0.8
	Fasting period/day >14 – 15 hours	0.7	0.5
	Fasting period/day >15 – 16 hours	0.9	0.4
**Trimester exposure analysis**	Model 1*		
	Never exposed to Ramadan	Ref.	
	1st Trimester exposure	0.2	0.8
	2nd Trimester exposure	1.3	0.2
	3rd Trimester exposure	1.9	0.06
	Model 2§		
	Never exposed to Ramadan	Ref.	
	1st Trimester exposure	-0.8	0.5
	2nd Trimester exposure	0.4	0.7
	3rd Trimester exposure	1.2	0.2

* Unadjusted model

§Adjusted for gestational age at delivery, gender, ethnicity, maternal age, mother weight gain, patient weight before pregnancy, and hypertensive disorders of pregnancy.
